# Supplementary figures and images for: An unbiased high‐throughput drug screen reveals a potential therapeutic vulnerability in the most lethal molecular subtype of pancreatic cancer
Source: Mol Oncol. 2020 Jul 4;14(8):1800–16. doi: 10.1002/1878-0261.12743 (PMC7400780; doi:10.1002/1878-0261.12743)

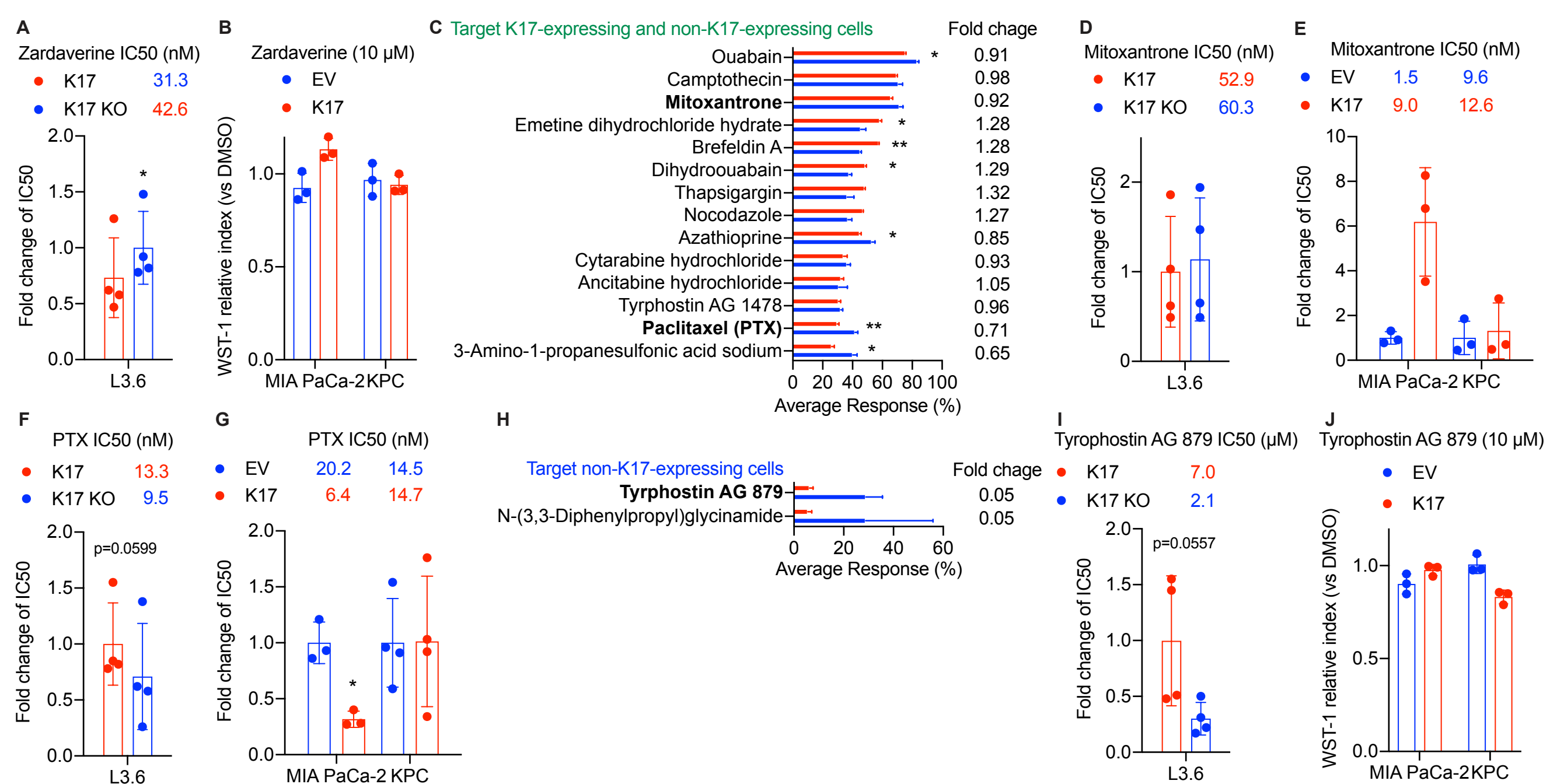

Supplement: Supplementary file 1 — Fig. S1. Validation of compounds in other categories. (A, B) Validation of Zardaverine in L3.6 (A) and in MIA PaCa‐2 and KPC cell line models (B). IC50 values, fold change of IC50 or cell viability (WST‐1 relative index) are shown. (C) Drugs targeting both L3.6 K17 expressing and KO cells from the Screen and the Counterscreen are listed. Fold change of average response rate are shown (mean ± SEM). (D, E) Validation of Mitoxantrone in L3.6 (D) and in MIA PaCa‐2 and KPC cell line models (E). IC50 values and fold change of IC50 are shown. (F, G) Validation of PTX in L3.6 (F) and in MIA PaCa‐2 and KPC cell line models (G). IC50 values and fold change of IC50 are shown. (H) Drugs targeting L3.6 K17 KO cells from the Screen and the Counterscreen are listed. Fold change of average response rate are shown (mean ± SEM). (I, J) Validation of Tyrophostin AG879 in L3.6 (I) and in MIA PaCa‐2 and KPC cell line models (J). IC50 values, fold change of IC50 or cell viability (WST‐1 relative index) are shown. Data are shown in mean ± SD. *P < 0.05, **P < 0.01, n = 3–4. Student's t‐test. [file MOL2-14-1800-s001.pdf]

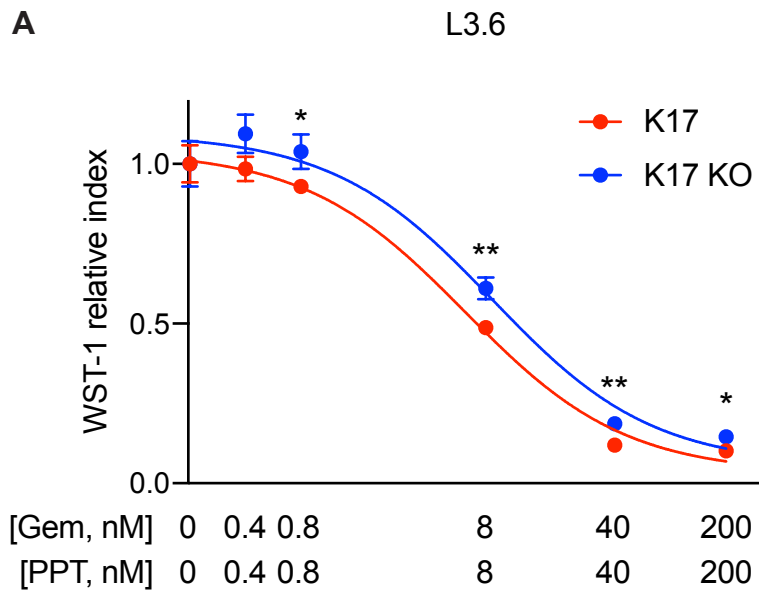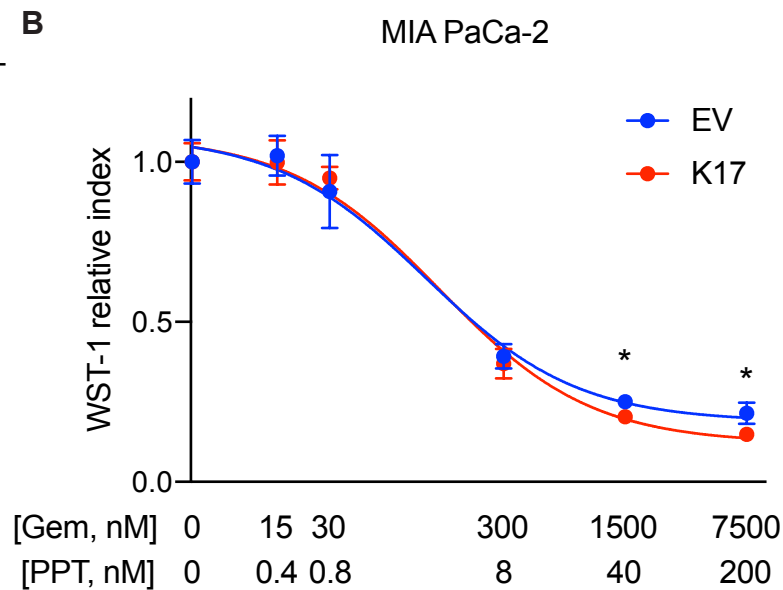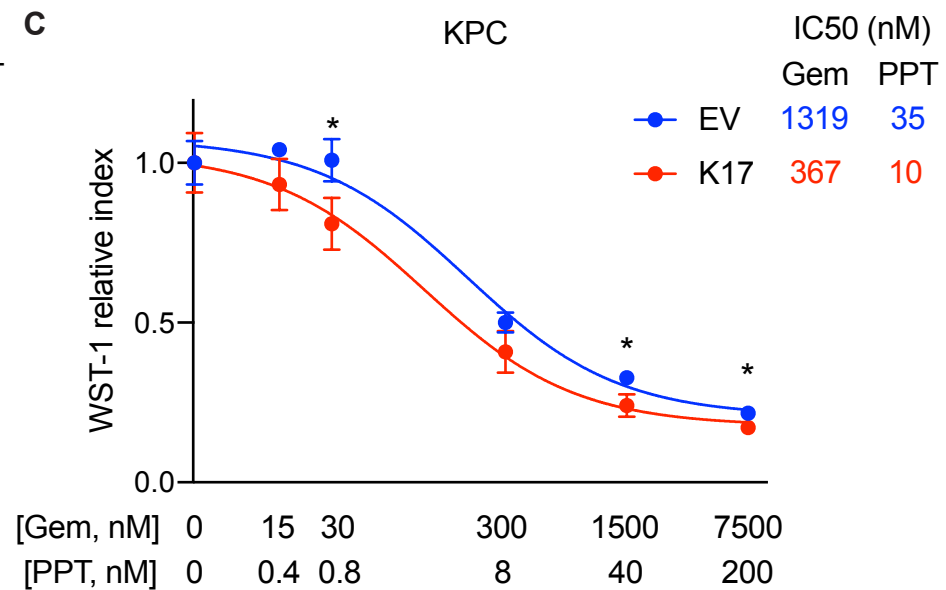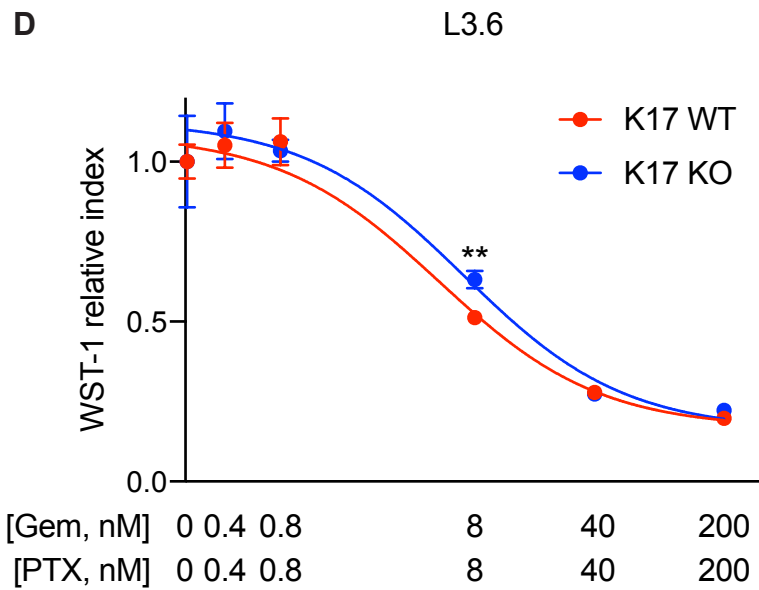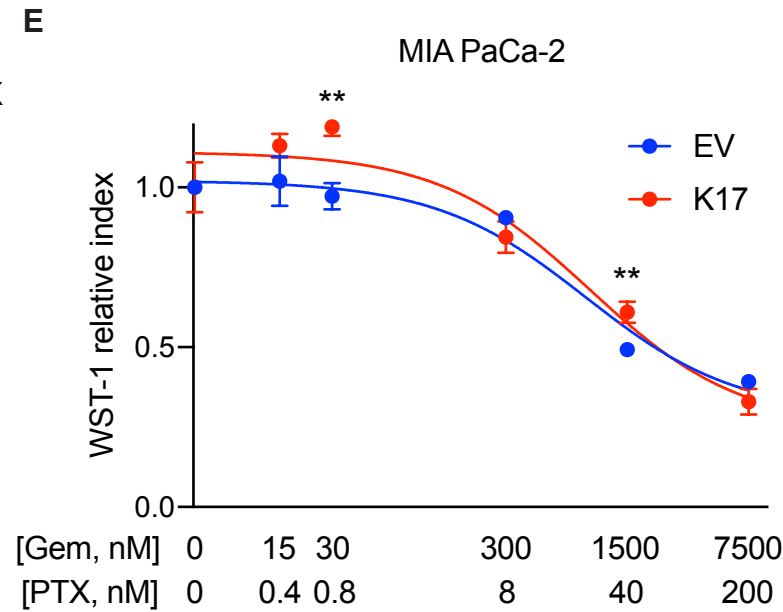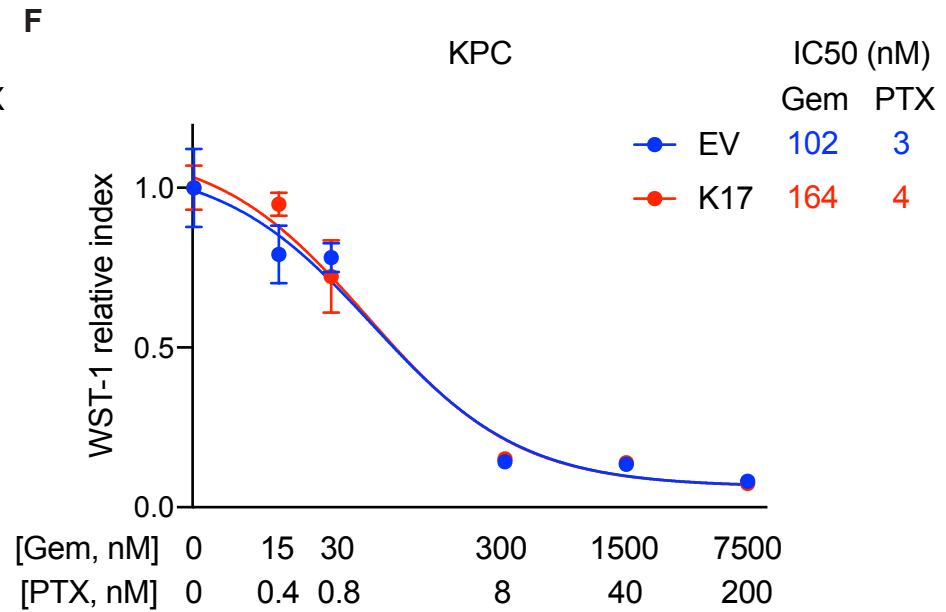

Supplement: Supplementary file 2 — Fig. S2. K17 expressing cells show lower cell viability than non‐K17 expressing cells under treatment of PPT and Gem, but no obvious difference is found in PTX and Gem. (A–C) The dose‐response curves of PPT combined with Gem were shown in L3.6 (A), MIA PaCa‐2 (B) and KPC (C) cell line models. The predicted IC50 of PPT + gem in each cell line were listed. (D–F) The dose‐response curves of PTX combined with Gem were shown in L3.6 (D), MIA PaCa‐2 (E) and KPC (F) cell line models. The predicted IC50 of PPT + gem in each cell line were listed. Data are shown in mean ± SD. *P < 0.05, **P < 0.01, ***P < 0.001, n = 3. Student's t‐test. [file MOL2-14-1800-s002.pdf]

**A**

EV

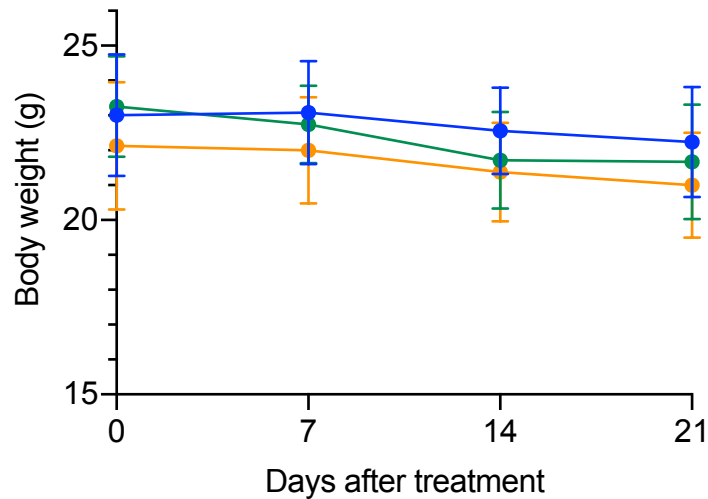**B**

K17

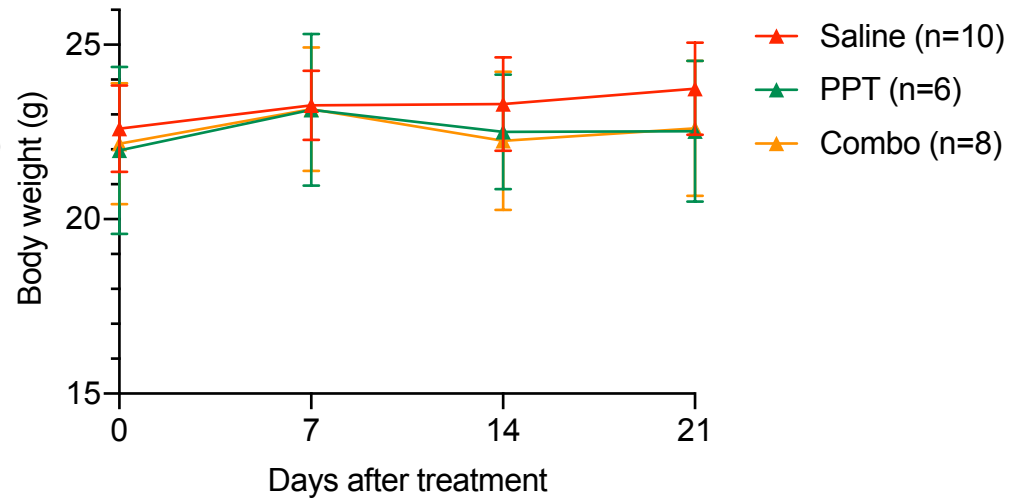

Supplement: Supplementary file 3 — Fig. S3. The mice tolerated all the treatments without significant body weight differences in the study of Gem combined with PPT. (A, B) Body weight of mice in each treatment group was shown in KPC EV (A) and K17 (B) tumors. [file MOL2-14-1800-s003.pdf]

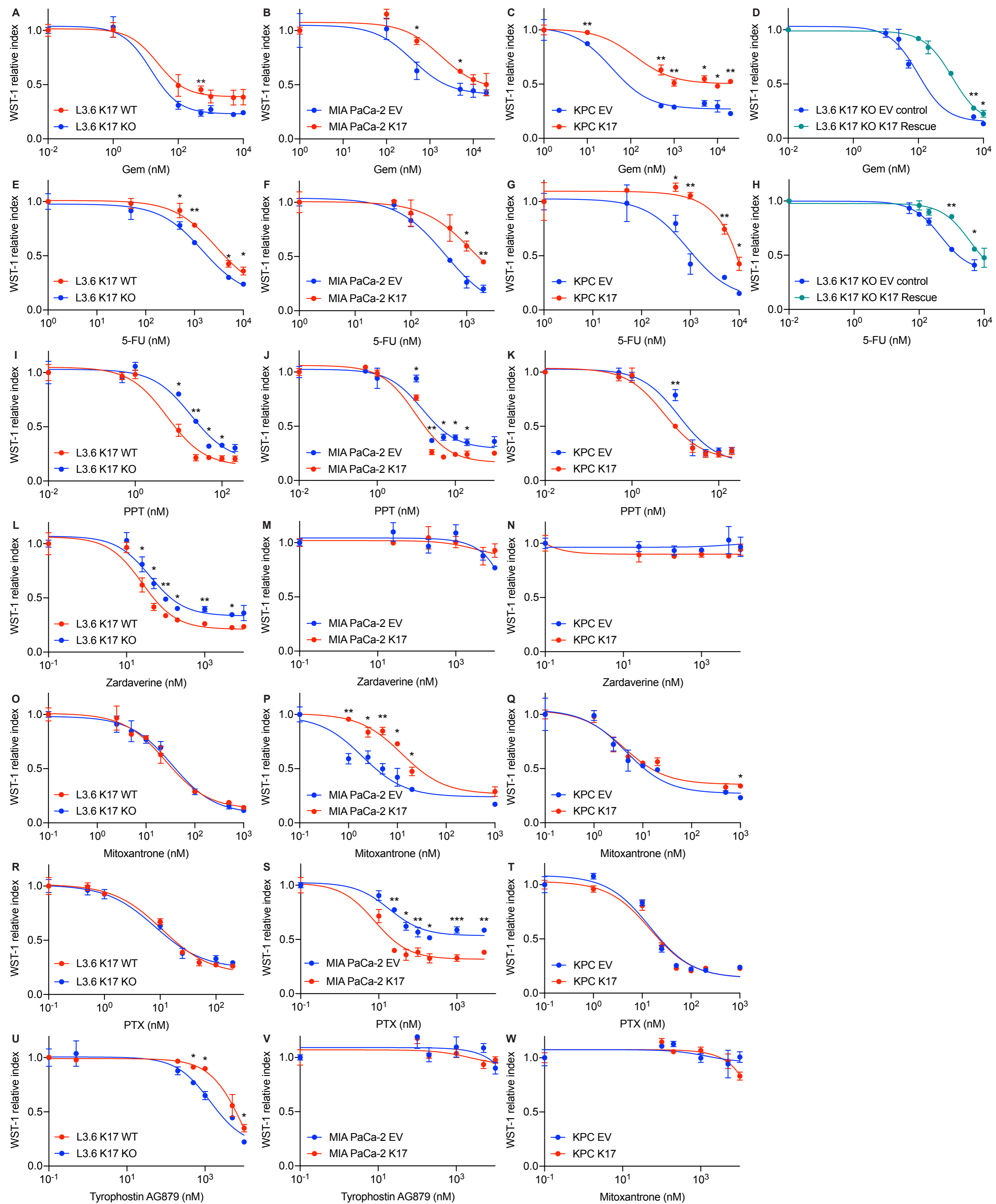

Supplement: Supplementary file 4 — Fig. S4. Representative dose response curves of each tested drugs. (A–D) Gem treatment in L3.6 K17 LOF cell line model (A), MIA PaCa‐2 (B) and KPC (C) K17 GOF cell line models, and L3.6 K17 Rescue cell line model (D). (E–H) 5‐FU treatment in L3.6 K17 LOF cell line model (E), MIA PaCa‐2 (F) and KPC (G) K17 GOF cell line models, and L3.6 K17 Rescue cell line model (H). (I–K) PPT treatment in L3.6 K17 LOF cell line model (I), MIA PaCa‐2 (J) and KPC (K) K17 GOF cell line models. (L–N) Zardaverine treatment in L3.6 K17 LOF cell line model (L), MIA PaCa‐2 (M) and KPC (N) K17 GOF cell line models. (O–Q) Mitoxantrone treatment in L3.6 K17 LOF cell line model (O), MIA PaCa‐2 (P) and KPC (Q) K17 GOF cell line models. (R–T) PTX treatment in L3.6 K17 LOF cell line model (R), MIA PaCa‐2 (S) and KPC (T) K17 GOF cell line models. (U–W) Tyrophostin AG879 treatment in L3.6 K17 LOF cell line model (U), MIA PaCa‐2 (V) and KPC (W) K17 GOF cell line models. Data was shown in mean ± SD. *P < 0.5, **P < 0.01, ***P < 0.001, n = 3. Student's t‐test. Fig. S4. Representative dose response curves of each tested drugs. [file MOL2-14-1800-s004.pdf]
